# Calculation of the Cost of Student Education: A Case Study of Shiraz Medical School

**DOI:** 10.30476/jamp.2020.86837.1271

**Published:** 2021-04

**Authors:** MOHAMMAD NAMAZI, SAHAR ZARE

**Affiliations:** 1 Department of Accounting, Shiraz University, Shiraz, Iran

**Keywords:** Activity-based costing, Accounting, Financial management, Medical school

## Abstract

**Introduction:**

Given the importance of the education system in the health sector and the necessity to calculate the cost in this sector, this study aimed to calculate the cost of education for health students in Shiraz Medical School, using activity-based costing (ABC).

**Methods:**

This study was conducted in Shiraz University of Medical Sciences in 2015-2016, considering the cost structure of the education department. The data required for the present research study was gathered from accrual accounting system, payroll system, educational deputy system called SAMA, list of paid salaries, information received from medical school such as exact position of individuals and course credits assigned to teachers, interviews conducted at the university headquarter in order to determine the appropriate indicators for allocating the costs, and interviewing clinical and non-clinical teachers to calculate the cost of training in these fields.

**Results:**

The findings indicated that the cost of training in general accounted for 70% of the total cost of student education. PhD in Medical Ethics, Assistant of Radiotherapy and Ph.D. in Pharmacology turned out to posit the highest cost for each student respectively, while MPH, Master of Medical Engineering, and Master of Electronic Medicine Education had the lowest cost for each student, respectively. The cost in all fields is more than the per capita cost of student education paid to the university.

**Conclusion:**

Authorities should focus on controlling and reducing the cost of training, which is the main component of the costs. Factors such as the number of students in each field, degree, and type of field are effective in the costs of education. Hence, in order to allocate the budget more equitably, costs of education for each field calculated by ABC should be based on allocating the funds to the university.

## Introduction

The health sector throughout the world is under a lot of pressure to improve its services, control costs, and manage its budget. Thus, in response to chronic financial constraints, management accounting tools and costing systems are utilized ( 1 ). University officials have also recognized the importance of management techniques for the management of universities and have noticed the growing need for an efficient accounting system for calculating the cost due to the reduction in available financial resources ( 2 ). Therefore, the issue of student and course costs has been the focus of attention both in research and practice ( 3 ).

Due to superiority of activity-based costing (ABC) over traditional cost accounting (TCA) system in decision-making ( 4 - 6 ) and subsequently ABC's significant impact on performance improvement as well as its benefits for budgeting levels, financial decision-making and strategic decision-making ( 7 , 8 ), the number of organizations implementing ABC is growing ( 9 ). Krishnan ( 10 ), among others, also indicates that ABC helps educational institutions improve their performance by accurately calculating the cost per student and by identifying nonvalue-added activities. Therefore, ABC can be utilized as a powerful tool to solve various university management problems in educational affairs. Much research has been conducted on the calculation of ABC in universities ( 11 - 14 ). These studies reveal how ABC is applied and what benefits it could have for universities ( 15 ). ABC allows university administrators to understand the factors influencing teaching in general and each course in particular, and this understanding is important in the decision-making process. For example, a university can increase its productivity by reconsidering its activities with a new and sensitive view of costs ( 16 ). It is worth noting that the full implementation of the ABC model depends on the understanding of all people working at the university about the benefits of this model, as well as a change in their view towards the cost management ( 17 ).

Some previous studies also have calculated the student costs using the ABC method in Iran ( 18 - 26 ). However, no research has so far been conducted in Iran regarding the calculation of the cost of clinical disciplines. The present study has provided some important advantages and strengths compared to previous research studies. This study is the first attempt to provide a model in this field. In addition, previous research has ignored the costs incurred to support the colleges by the University of Medical Sciences headquarters. Besides, since the information required to allocate to all faculties is considered in the present study, allocating headquarter costs to faculties will be attempted more accurately. Also, previous research studies failed to make a distinction between the salaries of professors as faculty members and their receipts as their activity in hospitals, and duty units with or without job positions have ignored the calculation of the cost allocated for salaries of professors.

Therefore, despite the benefits of ABC and its applications in many countries, the importance of adopting the ABC system in the health sector, especially in the clinical sector, has not yet been well recognized in Iran. Therefore, the cost of education in this section has not been determined accurately. As a result, university management in the health sector is struggling with the following issues: What are the cost patterns? What is the real cost of a student in clinical and non-clinical courses? How much tuition and budget is needed to effectively and efficiently manage these sectors? And how can management cope with the rising costs of health care and improve university performance? The aim of this study was to respond to the preceding enquiries. The ultimate goal of this article is to implement the ABC system in medical school, which includes both clinical and non-clinical courses, in order to reduce the difference between the practice and theory of using costing systems.

## Methods

The present study is a quantitative research which falls within the category of applied-developmental research in terms of research type and direction, and is considered as a survey with regard to research method. All fields of Shiraz Medical School constituted the statistical population of this study. The implementation of ABC varies in different areas such as education, services, trade, and manufacturing, and requires specific features in each area ( 27 ). According to the five steps provided for the implementation of ABC in universities by Amizawati et al. ( 28 ) and considering the cost structure of the education department in Shiraz Medical School, this study employed ABC system in order to benefit from its advantages using the information of the 2015-2016 academic year.

### Step 1: Identifying resources

**Figure 1:**
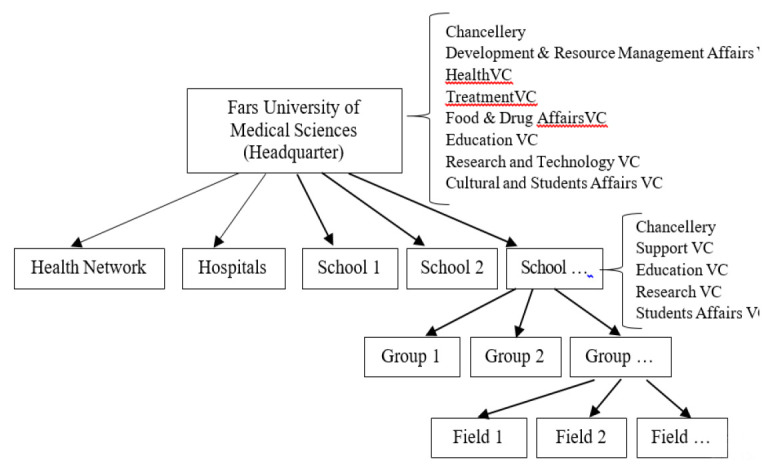
Overview of the Structure of the Health Sector

Figure 1 shows the general structure of Fars University of Medical Sciences as well as general overview of the schools and the groups and fields of each school. Information on the costs incurred in faculties and headquarter (except for the cost of salaries and benefits) was gathered from the "accrual system", information on salaries and benefits of the faculty members and the staff of both the headquarter and the faculty itself from the "salary system", information on the tuition fees paid from "Forms of tuition fees" paid by Educational deputy, and information on expenses incurred for research matters such as books, articles, etc. from "Research Deputy Documents". Also, educational information such as the number of courses taught by each teacher, number of students in each class, type of each course including degree, field, being theoretical or practical was collected from the educational deputy system called ‘SAMA’.

### Step 2: Identifying resource drivers

At the headquarter level, the expenses of Vice Chancellery for Health, Treatment, and Food & Drug Affairs, which provide most of their services to the health sector, have been eliminated. Due to the fact that no research has ever been conducted in Iran to allocate headquarter costs to subsidiary units in order to calculate the cost in the health sector, appropriate drivers to allocate the costs incurred in the headquarter to subsidiary units (like hospitals, faculties, etc.) were determined based on the information received from the interviews with the experts in different Vice Chancelleries: 1. “Education” and “Cultural and Students Affairs” Vice Chancelleries: Allocation to faculties based on the number of students in each faculty; 2. “Research and Technology” Vice Chancellery: Allocation to faculties based on the number of students (50%) and the number of faculty members (50%) of each faculty; and 3. “Chancellery” and “Development & Resource Management Affairs” Vice Chancellery: Allocation to all subordinate units (including hospitals, faculties, etc.) based on unit credits (40%), number of units (20%), and number of staff (40%). The preceding indicators are shown in Table 1.

**Table 1:** Drivers to Allocate the Costs Incurred in the Headquarter

Vice Chancelleries	Units	Cost Drivers
Education	Faculties	The number of students
Cultural and Students Affairs		
Research and Technology	Faculties	The number of students (50%)
		The number of Faculty Members (50%)
Chancellery	All Subordinate Units	Unit Credits (40%)
Development & Resource Management Affairs		Number of Units (20%)
		Number of Staff (40%)

School expenses during the academic year are represented in Table 2. As shown in Table 2, at the college level, most of the expenses (about 81% of total expenses for medical school) incurred in the education department are related to the salaries paid to the faculty members and the staff, and it is not cost-effective to pursue other expenses due to the low importance of the amount. Therefore, in the present study, all expenses incurred in faculty except salaries (such as water and electricity expenses) were considered together.

**Table 2:** School Expenses During the Academic Year

Account title	Amount(Rials)*	Percentage of total Expenses
Salaries and Benefits		
Salaries of Teachers	599,182,690,870	49.47160%
Salaries of Medical Assistants	224,591,965,137	18.54347%
Tuition fee	14,602,292,994	1.20564%
Staff Salaries	141,286,750,535	11.66536%
Total Salaries and Benefits	979,663,699,536	80.88607%
Other Expenses		
Use of goods and services:		
Mission and staff transfer costs	2,005,440,181	0.16558%
Transportation and communication costs	362,285,538	0.02991%
The cost of maintaining and repairing other assets	2,451,970,555	0.20245%
The cost of maintaining and repairing office equipment	2,135,723,410	0.17634%
Printing, buying magazines and press	231,036,100	0.01908%
Imaging and advertising	9,135,000	0.00075%
Ceremonies	3,037,552,967	0.25080%
Bank charges	895,250	0.00007%
Water, electricity and fuel	2,008,847,600	0.16586%
Consumable materials and supplies	17,895,471,321	1.47754%
Educational and Research Expenses	561,281,148	0.04634%
Fee for performing contract services	6,588,174,132	0.54395%
Social welfare:		
Welfare benefits for government employees	688,113,975	0.05681%
Student welfare assistance	160,303,668,574	13.23549%
Other Social Welfare Expenses	46,685,000	0.00385%
Other Expenses:		
Other Expenses	2,388,885,322	0.19724%
Group Sending aids	3,247,653,247	0.26814%
Depreciation Expenses of Assets:		
Depreciation Expenses of Building	17,154,274,968	1.41635%
Depreciation Expenses of Installations	94,649,493	0.00781%
Depreciation Expenses of equipment and machinery	6,911,783,669	0.57067%
Depreciation Expenses of Technical tools and equipment	9,741,345	0.00080%
Depreciation Expenses of	35,592,530	0.00294%
Depreciation Expenses of	3,331,849,697	0.27509%
Depreciation Expenses of	552,334	0.00005%
Other Expenses	231,501,263,351	19.11393%
Total Expenses	1,211,164,962,886	

* In order to convert the amount of this column to dollars, each amount could be divided by 35000, the approximate average exchange rate dominated at that time.

In order to accurately track the cost of salaries, the position of all individuals was precisely determined, and the cost of salaries of all employees and the payment of faculty members relevant to their job positions was allocated to the relevant position. Considering duty units with or without job positions, we separated the tuition fees of the professors with job positions at the academia. Therefore, the cost share related to the professor's position was separated from the cost share related to the teaching of each professor and was allocated to the relevant position and the number of courses taught, respectively.

### Step 3: Identifying activities

According to the four cost levels in the ABC system set out by Cooper and Kaplan ( 29 ), the course credit was defined as a "Unit" and the costs were classified as follows: UL: “Course credit cost level” like tuition fees for teaching the relevant course, BL: “Department cost level” such as the head of department's salary for their position in the department and the department secretary's fee; PL: “School cost level” such as expenses incurred in the faculty except salaries, which include water and electricity expenses and salaries of people who are related to all students. These people working in the faculties include the dean, vice-chancellor of the faculty, etc. Therefore, this cost level also includes expenses such as the salary received by the dean of the faculty for his/her position, the fee for the dean’s secretary, and the fee for the security guards of the faculty; FL: “Headquarter cost level” such as expenses incurred in “Chancellery” and “Education”, “Cultural and Students Affairs”, “Research and Technology”, and “Development & Resource Management Affairs” Vice Chancelleries (as determined in step 2).

### Step 4: Identifying activity drivers

The headquarter cost level and the faculty cost level are allocated in proportion to the number of students in the faculty, and the department cost level is allocated in proportion to the number of students in the respective department. For determining the tuition fees for each course, first the tuition fee for each class is determined and then the tuition fee for each student is calculated based on the number of students in each class. 

### Step 5: Calculating the cost of the products

The overhead cost was calculated from the total share of each student from the costs of headquarters, faculty vice chancelleries, faculty department, and other faculty expenses. Then, given that these costs are calculated for one academic year, the total overhead cost is calculated by considering the length of each field, as presented in Table 3.

**Table 3:** Cost of Overhead (Figures in Rials)*

Group	Code	Degree	Field	Each Student's Share of the Costs of						Overhead Cost for a Year(OC)	Overhead Cost Considering the Length of Each Fields(TOC)
				Headquarter (HC)		School's Sallaries (SC)		School's Other Costs (AC)			
				Amount	%	Amount	%	Amount	%		
General Practitioner	12104	General	General Practitioner	81,292,296	45%	22,821,206	13%	74,701,924	42%	178,815,426	1,251,707,979
Anatomy	12585	Master	Anatomy	81,292,296	28%	135,981,328	47%	74,701,924	26%	291,975,548	583,951,095
Anatomy	12586	Ph.D.	Anatomy	81,292,296	28%	135,981,328	47%	74,701,924	26%	291,975,548	1,313,889,965
Medical Ethics	12356	Ph.D.	Medical Ethics	81,292,296	9%	779,158,516	83%	74,701,924	8%	935,152,736	4,208,187,312
Biostatistics	12545	Master	Biostatistics	81,292,296	31%	109,652,623	41%	74,701,924	28%	265,646,843	531,293,685
Biostatistics	12566	Ph.D.	Biostatistics	81,292,296	31%	109,652,623	41%	74,701,924	28%	265,646,843	1,195,410,792
Parasitology and Mycology	12595	Master	Parasitology	81,292,296	30%	112,444,730	42%	74,701,924	28%	268,438,950	536,877,900
Parasitology and Mycology	12075	Master	Mycology	81,292,296	30%	112,444,730	42%	74,701,924	28%	268,438,950	536,877,900
Parasitology and Mycology	12596	Ph.D.	Parasitology	81,292,296	30%	112,444,730	42%	74,701,924	28%	268,438,950	1,207,975,275
Immunology	12155	Master	Immunology	81,292,296	25%	167,046,151	52%	74,701,924	23%	323,040,371	646,080,742
Immunology	12156	Ph.D.	Immunology	81,292,296	25%	167,046,151	52%	74,701,924	23%	323,040,371	1,453,681,670
Bacteriology-Virology	12135	Master	Microbiology	81,292,296	26%	162,145,296	51%	74,701,924	23%	318,139,516	636,279,032
Bacteriology-Virology	12097	Master	Virology	81,292,296	26%	162,145,296	51%	74,701,924	23%	318,139,516	636,279,032
Bacteriology-Virology	12136	Ph.D.	Bacteriology	81,292,296	26%	162,145,296	51%	74,701,924	23%	318,139,516	1,431,627,821
Clinical Biochemistry	12165	Master	Clinical Biochemistry	81,292,296	27%	140,362,240	47%	74,701,924	25%	296,356,460	592,712,920
Clinical Biochemistry	12166	Ph.D.	Clinical Biochemistry	81,292,296	27%	140,362,240	47%	74,701,924	25%	296,356,460	1,333,604,070
Medical-genetics	12565	Master	Medical-genetics	81,292,296	11%	587,900,200	79%	74,701,924	10%	743,894,420	1,487,788,840
Pharmacology	12201	Ph.D.	Pharmacology	81,292,296	14%	406,905,327	72%	74,701,924	13%	562,899,547	2,533,047,960
Medical Physic and Engineering	12055	Master	Medical Physic	81,292,296	33%	89,514,649	36%	74,701,924	30%	245,508,869	491,017,737
Medical Physic and Engineering	12065	Master	Medical Engineering	81,292,296	33%	89,514,649	36%	74,701,924	30%	245,508,869	491,017,737
Physiology	12175	Master	Physiology	81,292,296	26%	161,985,322	51%	74,701,924	23%	317,979,542	635,959,083
Physiology	12176	Ph.D.	Physiology	81,292,296	26%	161,985,322	51%	74,701,924	23%	317,979,542	1,430,907,937
MPH	12018	MPH	MD-MPH	81,292,296	43%	31,795,112	17%	74,701,924	40%	187,789,332	375,578,664
Pathology	12227	Assistant	Pathology	81,292,296	45%	22,821,206	13%	74,701,924	42%	178,815,426	715,261,702
Urology	12124	Assistant	Urology	81,292,296	44%	30,463,695	16%	74,701,924	40%	186,457,915	745,831,659
Urology	129011	Fellowship	Endourology	81,292,296	44%	30,463,695	16%	74,701,924	40%	186,457,915	279,686,872
Urology	12113	Fellowship	Uro-Oncology	81,292,296	44%	30,463,695	16%	74,701,924	40%	186,457,915	279,686,872
Neurology	12697	Assistant	Neurology	81,292,296	40%	48,811,376	24%	74,701,924	36%	204,805,596	819,222,385
Anesthesiology	12117	Master	Circulatory Technology	81,292,296	43%	34,800,986	18%	74,701,924	39%	190,795,206	381,590,412
Anesthesiology	12217	Assistant	Anesthesiology	81,292,296	44%	28,751,166	16%	74,701,924	40%	184,745,386	738,981,545
Anesthesiology	129511	Fellowship	Abdominal Organ Transplantation Anesthesia	81,292,296	44%	28,751,166	16%	74,701,924	40%	184,745,386	277,118,080
Anesthesiology	12412	Subspecialty	ICU	81,292,296	43%	34,342,154	18%	74,701,924	39%	190,336,374	380,672,748
Anesthesiology	129411	Fellowship	Cardiac Anesthesia	81,292,296	44%	28,751,166	16%	74,701,924	40%	184,745,386	277,118,080
Radiotherapy	12667	Assistant	Radiotherapy	81,292,296	38%	57,195,152	27%	74,701,924	35%	213,189,372	852,757,488
Social Medicine	12237	Assistant	Social Medicine	81,292,296	36%	68,822,630	31%	74,701,924	33%	224,816,850	674,450,549
Nuclear Medicine	12211	Assistant	Nuclear Medicine	81,292,296	37%	65,488,327	30%	74,701,924	34%	221,482,547	885,930,188
Dermatology	12247	Assistant	Dermatology	81,292,296	42%	39,392,437	20%	74,701,924	38%	195,386,657	781,546,628
Surgery	12267	Assistant	General Surgery	81,292,296	40%	47,608,066	23%	74,701,924	37%	203,602,286	814,409,142
Surgery	12105	Subspecialty	Plastic Surgery, Reconstructive, Burns	81,292,296	39%	53,199,053	25%	74,701,924	36%	209,193,273	627,579,820
Surgery	12312	Subspecialty	Vascular Surgery	81,292,296	39%	53,199,053	25%	74,701,924	36%	209,193,273	522,983,183
Surgery	129611	Fellowship	Colorectal Surgery	81,292,296	40%	47,608,066	23%	74,701,924	37%	203,602,286	305,403,428
Surgery	12204	Fellowship	Liver Transplantation	81,292,296	40%	47,608,066	23%	74,701,924	37%	203,602,286	407,204,571
Surgery	12828	Subspecialty	Pediatric Surgery	81,292,296	39%	53,199,053	25%	74,701,924	36%	209,193,273	627,579,820
Surgery	12083	Fellowship	Laparoscopy	81,292,296	40%	47,608,066	23%	74,701,924	37%	203,602,286	305,403,428
Surgery	12053	Fellowship	CancerSurgery	81,292,296	40%	47,608,066	23%	74,701,924	37%	203,602,286	305,403,428
Neurosurgery	12337	Assistant	Neurosurgery	81,292,296	36%	67,819,825	30%	74,701,924	33%	223,814,044	1,119,070,222
Neurosurgery	12128	Fellowship	Spine Surgery	81,292,296	36%	67,819,825	30%	74,701,924	33%	223,814,044	895,256,178
Ophthalmology	12277	Assistant	Ophthalmology	81,292,296	43%	33,733,530	18%	74,701,924	39%	189,727,750	758,911,001
Ophthalmology	129111	Fellowship	Eye pathology	81,292,296	43%	33,733,530	18%	74,701,924	39%	189,727,750	284,591,625
Ophthalmology	12153	Fellowship	Cornea and External Eye Diseases	81,292,296	43%	33,733,530	18%	74,701,924	39%	189,727,750	284,591,625
Ophthalmology	12073	Fellowship	Pediatric Ophthalmology and Eye Aberrations	81,292,296	43%	33,733,530	18%	74,701,924	39%	189,727,750	284,591,625
Ophthalmology	12127	Fellowship	Retina	81,292,296	43%	33,733,530	18%	74,701,924	39%	189,727,750	284,591,625
Internal-Medicine	12287	Assistant	Internal Diseases	81,292,296	44%	29,227,261	16%	74,701,924	40%	185,221,481	740,885,923
Internal-Medicine	12126	Assistant	Internal-Cardiology	81,292,296	44%	29,227,261	16%	74,701,924	40%	185,221,481	740,885,923
Internal-Medicine	12768	Subspecialty	Endocrine and Metabolism Glands	81,292,296	43%	34,818,249	18%	74,701,924	39%	190,812,468	381,624,937
Internal-Medicine	12948	Subspecialty	Adult Blood and Cancer	81,292,296	43%	34,818,249	18%	74,701,924	39%	190,812,468	572,437,405
Internal-Medicine	12212	Subspecialty	Adult Digestive and Liver	81,292,296	43%	34,818,249	18%	74,701,924	39%	190,812,468	381,624,937
Internal-Medicine	12968	Subspecialty	Nephrology (Adult Kidney)	81,292,296	43%	34,818,249	18%	74,701,924	39%	190,812,468	381,624,937
Internal-Medicine	12738	Subspecialty	Lung Diseases	81,292,296	43%	34,818,249	18%	74,701,924	39%	190,812,468	381,624,937
Internal-Medicine	12748	Subspecialty	Rheumatology	81,292,296	43%	34,818,249	18%	74,701,924	39%	190,812,468	381,624,937
Radiology	12867	Assistant	Radiology	81,292,296	45%	22,847,900	13%	74,701,924	42%	178,842,120	715,368,480
Radiology	12023	Fellowship	Interventional Radiology	81,292,296	45%	22,847,900	13%	74,701,924	42%	178,842,120	178,842,120
Psychiatry	12307	Assistant	Psychiatry	81,292,296	36%	70,106,500	31%	74,701,924	33%	226,100,720	904,402,878
Obstetrics & Gynecology	12317	Assistant	Obstetrics & Gynecology	81,292,296	42%	36,438,879	19%	74,701,924	39%	192,433,099	769,732,395
Obstetrics & Gynecology	12033	Fellowship	Perinatology	81,292,296	42%	36,438,879	19%	74,701,924	39%	192,433,099	288,649,648
Obstetrics & Gynecology	128911	Fellowship	Women's Oncology	81,292,296	42%	36,438,879	19%	74,701,924	39%	192,433,099	288,649,648
Obstetrics & Gynecology	129811	Fellowship	IVF	81,292,296	42%	36,438,879	19%	74,701,924	39%	192,433,099	288,649,648
Obstetrics & Gynecology	12043	Fellowship	Laparoscopy	81,292,296	42%	36,438,879	19%	74,701,924	39%	192,433,099	288,649,648
Emergency Medicine	12111	Assistant	Emergency Medicine	81,292,296	42%	37,987,641	20%	74,701,924	39%	193,981,861	581,945,584
Traditional Medicine	12186	Ph.D.	Traditional Medicine	81,292,296	24%	189,414,419	55%	74,701,924	22%	345,408,639	1,554,338,877
Physical Medicine & Rehabilitation	12257	Assistant	Physical Medicine & Rehabilitation	81,292,296	39%	50,144,914	24%	74,701,924	36%	206,139,134	618,417,401
Cardiology	12657	Assistant	Cardiology Diseases	81,292,296	42%	38,210,197	20%	74,701,924	38%	194,204,417	776,817,668
Pediatrics	12197	Assistant	Pediatric Diseases	81,292,296	42%	36,215,780	19%	74,701,924	39%	192,210,000	768,840,000
Pediatrics	12978	Subspecialty	Pediatric Cancer and Blood	81,292,296	41%	41,806,768	21%	74,701,924	38%	197,800,988	593,402,963
Pediatrics	12838	Subspecialty	Children Digestive	81,292,296	41%	41,806,768	21%	74,701,924	38%	197,800,988	395,601,976
Pediatrics	12798	Subspecialty	Children Infectious	81,292,296	41%	41,806,768	21%	74,701,924	38%	197,800,988	395,601,976
Pediatrics	12778	Subspecialty	Pediatric Cardiology	81,292,296	41%	41,806,768	21%	74,701,924	38%	197,800,988	593,402,963
Pediatrics	12198	Subspecialty	Neonatal Medicine	81,292,296	41%	41,806,768	21%	74,701,924	38%	197,800,988	395,601,976
Pediatrics	12788	Subspecialty	Clinical Immunology and Allergy	81,292,296	41%	41,806,768	21%	74,701,924	38%	197,800,988	593,402,963
Pediatrics	12205	Fellowship	PICU	81,292,296	42%	36,215,780	19%	74,701,924	39%	192,210,000	288,315,000
Pediatrics	12123	Fellowship	Children Cardiology Intervention	81,292,296	42%	36,215,780	19%	74,701,924	39%	192,210,000	288,315,000
Pediatrics	12112	Subspecialty	Endocrine and Metabolism Glands of Children	81,292,296	41%	41,806,768	21%	74,701,924	38%	197,800,988	395,601,976
ENT	12327	Assistant	ENT	81,292,296	38%	55,936,905	26%	74,701,924	35%	211,931,125	847,724,500
MedicalEducation	12385	Master	Electronic Medicine Education	81,292,296	44%	28,871,025	16%	74,701,924	40%	184,865,245	369,730,490
Family Doctor	12311	Assistant	Family Doctor	81,292,296	36%	72,922,595	32%	74,701,924	33%	228,916,815	515,062,835
Orthopedics	12125	Assistant	Orthopedics	81,292,296	45%	22,821,206	13%	74,701,924	42%	178,815,426	715,261,702
Orthopedics	12093	Fellowship	Spine Surgery	81,292,296	45%	22,821,206	13%	74,701,924	42%	178,815,426	268,223,138
Orthopedics	12163	Fellowship	Pediatric Orthopedics	81,292,296	45%	22,821,206	13%	74,701,924	42%	178,815,426	268,223,138
Cardiology	12143	Fellowship	Adult Cardiology Intervention	81,292,296	45%	22,821,206	13%	74,701,924	42%	178,815,426	268,223,138
Cardiology	12658	Subspecialty	Cardiology Diseases	81,292,296	44%	28,412,193	15%	74,701,924	41%	184,406,413	737,625,653

* In order to convert the amount of this column to dollars, each amount could be divided by 35000, the approximate average exchange rate dominated at that time.

To calculate each student's share of the costs of teaching non-clinical courses, the following were conducted: 1. Determining the cost of teaching each course credit by a professor: “The tuition fee and benefits paid to a teacher for teaching” (includes the tuition fee of the faculty member minus payment for job position, added by tuition fee) divided by “the number of credits taught by each professor during the school year”, taking into account the coefficient of the degree, theoretical or practical, the type of course, the number of professors in each class. 2. Determining the cost of each student in each classroom: “The cost of teaching each class” (the cost of tuition fee paid to a specific teacher for a single course credit multiplied by the number of credits equivalent to the corresponding class taught by the same professor) divided by “the number of students in that class”. 3. Determining the cost of each course: The average cost per student for each course in different classes. 4. Determining the cost of education in each field: According to the syllabus of each field, the cost of courses that were taught in the academic year 2015-2016 was considered, and an estimate of the training cost of practical and theoretical credits for the whole field was studied.

**Figure 2:**
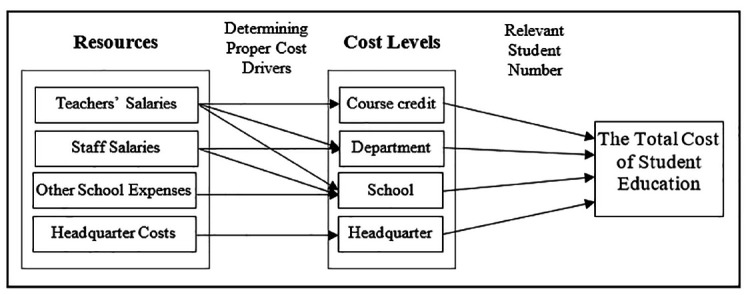
Model of the Cost of Student Education

Unlike non-clinical courses, clinical courses do not have a course credit and some do not have even a course code. Due to the fact that some teachers teach in both clinical and non-clinical fields, first the share of non-clinical courses was calculated according to the duty units of each professor as well as the number of credits taught in non-clinical courses. The rest of the professor's salaries were allocated to teaching clinical courses. Then, the clinical groups and subgroups and their corresponding professors were identified. The rotation of each clinical field was precisely determined. After carefully examining how to teach clinical courses and after interviewing the experts of these courses, it was concluded that the amount of service provided to students, in addition to their routine, depends on the duration of attendance in the department, the number of students and their degree. In addition, given that the courses that require the operating room differed from those that do not, clinical professors believed that these disciplines should be considered separately. Finally, “the duration of attendance in the department (per month) * number of students” was considered as a criterion to measure the extent to which each student use and benefit from a professor's training of each main group and subgroup. In addition, since the university degree is also an effective factor in the use of professor training, it was determined (through a survey of clinical teachers) that the fields requiring an operating room be allocated teacher training of approximately 30%, 40% and 30%, and fields that do not require an operating room 26%, 33% and 41%, respectively, for general medicine, assistant and subspecialty or fellowship. Figure 2 indicates an overview of the model of the cost of student education.

The present study is the result of a doctoral dissertation conducted at Shiraz University, but the code of ethics is not common in the Ministry of Science, Research and Technology, and despite full observance of ethics in this research, it does not have a code of ethics.

## Results

The results of calculating the cost of students in all fields of Shiraz Medical School are presented in Table 4. In order to help in analysis and management, the average cost at different degrees has separately been presented to show the cost of training and the overhead, as well as the share of each in the total costs in percentage. Also, to help better compare the amount of the costs with each other, the rankings of each component of the cost are presented relative to all fields. In Table 5, the average cost values are presented separately for different degrees as well as the total average for all degrees. This Table also demonstrates the average of components and total costs by clinical and non-clinical disciplines.

**Table 4:** The Total Cost of Student Education (Figures in Rials)*

Group	Code	Degree	Field	Overhead Cost			Training Cost			Total Cost	
				Amount	Rank	%	Amount	Rank	%	Amount	Rank
General Practitioner	12104	General	General Practitioner	1,251,707,979	10	40%	1,865,267,270	27	60%	3,116,975,249	11
Anatomy	12585	Master	Anatomy	583,951,095	46	53%	515,131,564	80	47%	1,099,082,660	77
Anatomy	12586	Ph.D.	Anatomy	1,313,889,965	9	45%	1,576,524,662	38	55%	2,890,414,626	18
Medical Ethics	12356	Ph.D.	Medical Ethics	4,208,187,312	1	50%	4,171,214,201	4	50%	8,379,401,513	1
Biostatistics	12545	Master	Biostatistics	531,293,685	51	59%	363,208,689	87	41%	894,502,374	86
Biostatistics	12566	Ph.D.	Biostatistics	1,195,410,792	12	56%	930,389,889	66	44%	2,125,800,682	42
Parasitology and Mycology	12595	Master	Parasitology	536,877,900	49	53%	483,655,862	83	47%	1,020,533,761	82
Parasitology and Mycology	12075	Master	Mycology	536,877,900	50	60%	357,939,577	88	40%	894,817,477	85
Parasitology and Mycology	12596	Ph.D.	Parasitology	1,207,975,275	11	45%	1,447,895,607	42	55%	2,655,870,882	20
Immunology	12155	Master	Immunology	646,080,742	35	40%	964,687,629	64	60%	1,610,768,371	61
Immunology	12156	Ph.D.	Immunology	1,453,681,670	5	55%	1,174,017,281	54	45%	2,627,698,951	21
Bacteriology-Virology	12135	Master	Microbiology	636,279,032	37	57%	475,575,941	84	43%	1,111,854,972	75
Bacteriology-Virology	12097	Master	Virology	636,279,032	36	45%	780,221,144	71	55%	1,416,500,176	67
Bacteriology-Virology	12136	Ph.D.	Bacteriology	1,431,627,821	6	49%	1,474,723,838	40	51%	2,906,351,659	17
Clinical Biochemistry	12165	Master	Clinical Biochemistry	592,712,920	45	46%	703,892,844	77	54%	1,296,605,764	71
Clinical Biochemistry	12166	Ph.D.	Clinical Biochemistry	1,333,604,070	8	51%	1,276,396,071	49	49%	2,610,000,141	22
Medical-genetics	12565	Master	Medical-genetics	1,487,788,840	4	64%	847,625,495	68	36%	2,335,414,335	33
Pharmacology	12201	Ph.D.	Pharmacology	2,533,047,960	2	41%	3,685,759,992	5	59%	6,218,807,952	3
Medical Physic and Engineering	12055	Master	Medical Physic	491,017,737	54	50%	494,202,144	82	50%	985,219,881	83
Medical Physic and Engineering	12065	Master	Medical Engineering	491,017,737	55	68%	228,425,474	90	32%	719,443,211	89
Physiology	12175	Master	Physiology	635,959,083	38	58%	458,306,421	85	42%	1,094,265,504	78
Physiology	12176	Ph.D.	Physiology	1,430,907,937	7	46%	1,681,712,060	33	54%	3,112,619,998	12
MPH	12018	MPH	MD-MPH	375,578,664	68	53%	332,720,863	89	47%	708,299,527	90
Pathology	12227	Assistant	Pathology	715,261,702	32	21%	2,734,006,338	9	79%	3,449,268,040	9
Urology	12124	Assistant	Urology	745,831,659	26	43%	1,005,418,909	63	57%	1,751,250,568	57
Urology	129011	Fellowship	Endourology	279,686,872	83	11%	2,264,904,971	14	89%	2,544,591,843	25
Urology	12113	Fellowship	Uro-Oncology	279,686,872	84	12%	2,150,010,430	16	88%	2,429,697,303	30
Neurology	12697	Assistant	Neurology	819,222,385	19	30%	1,912,979,362	23	70%	2,732,201,747	19
Anesthesiology	12117	Master	Circulatory Technology	381,590,412	66	31%	849,293,195	67	69%	1,230,883,607	74
Anesthesiology	12217	Assistant	Anesthesiology	738,981,545	29	37%	1,273,964,067	51	63%	2,012,945,613	49
Anesthesiology	129511	Fellowship	Abdominal Organ Transplantation Anesthesia	277,118,080	86	19%	1,147,209,631	58	81%	1,424,327,710	66
Anesthesiology	12412	Subspecialty	ICU	380,672,748	67	21%	1,472,433,556	41	79%	1,853,106,304	55
Anesthesiology	129411	Fellowship	Cardiac Anesthesia	277,118,080	85	5%	5,185,809,243	3	95%	5,462,927,322	5
Radiotherapy	12667	Assistant	Radiotherapy	852,757,488	17	13%	5,778,924,683	1	87%	6,631,682,170	2
Social Medicine	12237	Assistant	Social Medicine	674,450,549	34	48%	739,240,103	75	52%	1,413,690,653	68
Nuclear Medicine	12211	Assistant	Nuclear Medicine	885,930,188	16	22%	3,079,910,766	8	78%	3,965,840,954	7
Dermatology	12247	Assistant	Dermatology	781,546,628	21	36%	1,392,987,935	45	64%	2,174,534,563	39
Surgery	12267	Assistant	General Surgery	814,409,142	20	14%	5,197,856,322	2	86%	6,012,265,464	4
Surgery	12105	Subspecialty	Plastic Surgery, Reconstructive, Burns	627,579,820	40	47%	707,381,906	76	53%	1,334,961,726	70
Surgery	12312	Subspecialty	Vascular Surgery	522,983,183	52	21%	1,973,466,962	21	79%	2,496,450,146	28
Surgery	129611	Fellowship	Colorectal Surgery	305,403,428	70	21%	1,167,124,056	55	79%	1,472,527,485	65
Surgery	12204	Fellowship	Liver Transplantation	407,204,571	56	43%	539,228,176	79	57%	946,432,747	84
Surgery	12828	Subspecialty	Pediatric Surgery	627,579,820	39	25%	1,927,390,219	22	75%	2,554,970,039	24
Surgery	12083	Fellowship	Laparoscopy	305,403,428	71	28%	798,822,159	70	72%	1,104,225,588	76
Surgery	12053	Fellowship	Cancer Surgery	305,403,428	72	35%	556,658,057	78	65%	862,061,486	87
Neurosurgery	12337	Assistant	Neurosurgery	1,119,070,222	13	44%	1,422,416,293	44	56%	2,541,486,516	26
Neurosurgery	12128	Fellowship	Spine Surgery	895,256,178	15	30%	2,073,368,899	18	70%	2,968,625,077	16
Ophthalmology	12277	Assistant	Ophthalmology	758,911,001	25	40%	1,148,537,442	56	60%	1,907,448,443	52
Ophthalmology	129111	Fellowship	Eye pathology	284,591,625	81	18%	1,323,210,874	47	82%	1,607,802,500	62
Ophthalmology	12153	Fellowship	Cornea and External Eye Diseases	284,591,625	79	12%	2,041,166,630	19	88%	2,325,758,255	34
Ophthalmology	12073	Fellowship	Pediatric Ophthalmology and Eye Aberrations	284,591,625	80	12%	2,016,021,814	20	88%	2,300,613,439	35
Ophthalmology	12127	Fellowship	Retina	284,591,625	82	27%	768,754,594	74	73%	1,053,346,219	80
Internal-Medicine	12287	Assistant	Internal Diseases	740,885,923	27	37%	1,248,769,119	52	63%	1,989,655,042	50
Internal-Medicine	12126	Assistant	Internal-Cardiology	740,885,923	28	40%	1,105,216,196	59	60%	1,846,102,119	56
Internal-Medicine	12768	Subspecialty	Endocrine and Metabolism Glands	381,624,937	63	17%	1,905,407,675	24	83%	2,287,032,611	36
Internal-Medicine	12948	Subspecialty	Adult Blood and Cancer	572,437,405	48	23%	1,897,086,277	25	77%	2,469,523,683	29
Internal-Medicine	12212	Subspecialty	Adult Digestive and Liver	381,624,937	64	19%	1,652,935,380	35	81%	2,034,560,317	48
Internal-Medicine	12968	Subspecialty	Nephrology (Adult Kidney)	381,624,937	62	15%	2,211,420,120	15	85%	2,593,045,057	23
Internal-Medicine	12738	Subspecialty	Lung Diseases	381,624,937	61	10%	3,533,055,816	6	90%	3,914,680,753	8
Internal-Medicine	12748	Subspecialty	Rheumatology	381,624,937	65	23%	1,274,216,265	50	77%	1,655,841,201	60
Radiology	12867	Assistant	Radiology	715,368,480	31	48%	775,183,226	73	52%	1,490,551,706	64
Radiology	12023	Fellowship	Interventional Radiology	178,842,120	90	14%	1,073,274,159	62	86%	1,252,116,279	72
Psychiatry	12307	Assistant	Psychiatry	904,402,878	14	36%	1,599,880,090	36	64%	2,504,282,968	27
Obstetrics & Gynecology	12317	Assistant	Obstetrics & Gynecology	769,732,395	23	37%	1,335,932,581	46	63%	2,105,664,976	44
Obstetrics & Gynecology	12033	Fellowship	Perinatology	288,649,648	73	12%	2,087,578,167	17	88%	2,376,227,815	31
Obstetrics & Gynecology	128911	Fellowship	Women's Oncology	288,649,648	74	13%	1,871,056,677	26	87%	2,159,706,326	40
Obstetrics & Gynecology	129811	Fellowship	IVF	288,649,648	75	21%	1,077,015,442	61	79%	1,365,665,090	69
Obstetrics & Gynecology	12043	Fellowship	Laparoscopy	288,649,648	76	23%	960,608,560	65	77%	1,249,258,208	73
Emergency Medicine	12111	Assistant	Emergency Medicine	581,945,584	47	53%	511,028,109	81	47%	1,092,973,694	79
Traditional Medicine	12186	Ph.D.	Traditional Medicine	1,554,338,877	3	66%	798,914,788	69	34%	2,353,253,665	32
Physical Medicine & Rehabilitation	12257	Assistant	Physical Medicine & Rehabilitation	618,417,401	41	36%	1,096,723,833	60	64%	1,715,141,235	59
Cardiology	12657	Assistant	Cardiology Diseases	776,817,668	22	34%	1,478,455,379	39	66%	2,255,273,047	37
Pediatrics	12197	Assistant	Pediatric Diseases	768,840,000	24	24%	2,487,909,076	12	76%	3,256,749,077	10
Pediatrics	12978	Subspecialty	Pediatric Cancer and Blood	593,402,963	44	20%	2,447,557,485	13	80%	3,040,960,449	15
Pediatrics	12838	Subspecialty	Children Digestive	395,601,976	58	19%	1,717,735,552	31	81%	2,113,337,527	43
Pediatrics	12798	Subspecialty	Children Infectious	395,601,976	57	19%	1,740,636,660	29	81%	2,136,238,636	41
Pediatrics	12778	Subspecialty	Pediatric Cardiology	593,402,963	43	19%	2,496,432,297	11	81%	3,089,835,260	14
Pediatrics	12198	Subspecialty	Neonatal Medicine	395,601,976	59	19%	1,684,632,329	32	81%	2,080,234,304	46
Pediatrics	12788	Subspecialty	Clinical Immunology and Allergy	593,402,963	42	19%	2,497,503,535	10	81%	3,090,906,499	13
Pediatrics	12205	Fellowship	PICU	288,315,000	78	18%	1,296,748,470	48	82%	1,585,063,470	63
Pediatrics	12123	Fellowship	Children Cardiology Intervention	288,315,000	77	17%	1,436,454,786	43	83%	1,724,769,786	58
Pediatrics	12112	Subspecialty	Endocrine and Metabolism Glands of Children	395,601,976	60	19%	1,672,441,312	34	81%	2,068,043,288	47
ENT	12327	Assistant	ENT	847,724,500	18	21%	3,186,742,563	7	79%	4,034,467,062	6
Medical Education	12385	Master	Electronic Medicine Education	369,730,490	69	46%	440,742,151	86	54%	810,472,641	88
Family Doctor	12311	Assistant	Family Doctor	515,062,835	53	23%	1,738,930,930	30	77%	2,253,993,765	38
Orthopedics	12125	Assistant	Orthopedics	715,261,702	33	37%	1,207,283,641	53	63%	1,922,545,343	51
Orthopedics	12093	Fellowship	Spine Surgery	268,223,138	87	13%	1,817,102,826	28	87%	2,085,325,964	45
Orthopedics	12163	Fellowship	Pediatric Orthopedics	268,223,138	89	26%	776,473,897	72	74%	1,044,697,036	81
Cardiology	12143	Fellowship	Adult Cardiology Intervention	268,223,138	88	14%	1,596,933,326	37	86%	1,865,156,464	54
Cardiology	12658	Subspecialty	Cardiology Diseases	737,625,653	30	39%	1,148,493,873	57	61%	1,886,119,526	53

* In order to convert the amount of this column to dollars, each amount could be divided by 35000, the approximate average exchange rate dominated at that time.

**Table 5:** Descriptive Statistics for Component of Education Cost (Rials figures)*

Degree	ClinicalorNon-Clinical	Number	The Average ofOverhead Cost			The Average ofTraining Cost			The Average ofTotal Cost		Estimated Budget for Each Degree	
			Amount	Rank	%	Amount	Rank	%	Amount	Rank	BudgetAmount	Budget Difference with Cost
MPH	Non-Clinical	1	375,578,664	6	53%	332,720,863	7	47%	708,299,527	7	193,018,600	-515,280,927
Master	Non-Clinical	14	611,246,900	4	52%	568,779,152	6	48%	1,180,026,053	6	238,093,300	-941,932,753
Ph.D.	Non-Clinical	10	1,766,267,168	1	49%	1,821,754,839	4	51%	3,588,022,007	1	702,038,700	-2,885,983,307
General Practitioner	Clinical	1	1,251,707,979	2	40%	1,865,267,270	3	60%	3,116,975,249	2	1,092,060,200	-2,024,915,049
Assistant	Clinical	23	765,292,078	3	29%	1,889,491,172	1	71%	2,654,783,251	3	736,734,789	-1,918,048,461
Fellowship	Clinical	23	312,408,155	7	17%	1,566,327,645	5	83%	1,878,735,801	5	308,885,974	-1,569,849,827
Subspecialty	Clinical	18	485,534,450	5	20%	1,886,679,290	2	80%	2,372,213,740	4	474,691,883	-1,897,521,857
All Degrees		90	681,934,893		30%	1,575,805,608		70%	2,257,740,501			
Non-Clinical Field		25	1,063,828,278		50%	1,060,527,095		50%	2,124,355,373			
Clinical Field		65	535,052,822		23%	1,773,989,651		77%	2,309,042,473			

* In order to convert the amount of this column to dollars, each amount could be divided by 35000, the approximate average exchange rate dominated at that time.

## Discussion

The results of Table 4 show that the PhD in Medical Ethics, assistant of Radiotherapy and Ph.D. in Pharmacology turned out to have the highest cost for each student respectively, while MPH, Master of Medical Engineering, and Master of Electronic Medicine Education had the lowest cost for each student, respectively. In addition, assistant of Radiotherapy, assistant of General Surgery, Fellowship of Cardiac Anesthesia, respectively, demonstrated the highest, but Master of Medical Engineering, MPH, and Master of Mycology, respectively, had the lowest tuition fees per student. Furthermore, in terms of overhead prices, Ph.D. in Medical Ethics, Ph.D. in pharmacology, and Ph.D. in Traditional Medicine, respectively, had the highest cost of overhead, and fellowships of Interventional Radiology, Spine Surgery, Pediatric Orthopedics, and Adult Cardiology Intervention had the lowest overhead costs, respectively.

Table 5 shows the average cost at different degrees, separated into the average cost of education and overhead. In addition, according to the cost ranking, it is observed that the PhD., General Practitioner and Assistant had the highest costs, respectively. It seems that the most important reason for the higher cost of these degrees is the higher cost of overhead. According to the overhead ranking, it can be seen that these degrees have the highest amounts of overhead compared to other degrees. The main reason for the higher overhead amounts in these degrees is that they take longer than other degrees. As explained in the method section of this study, the final overhead cost is calculated for one academic year and is determined by the length of the course. Because the longer a student spends time at the university, the more ancillary services he or she will receive.

However, in general, the cost of training accounts for 70% of the total cost of student education, and the importance of paying attention to the cost of education is also highlighted. The share of the cost of education in the total cost of education for students in the degrees of fellowship (83%), subspecialty (80%), and assistant (71%) were higher than average. Factors such as the small number of students in these degrees compared to other degrees; the use of professors with higher academic degrees, which consequently costs more; and the use of equivalent credits in graduate programs can be among the factors influencing the higher cost of education. So far, no research study has been conducted on calculation of the cost of education in the field of clinical disciplines. Therefore, we compared the results of the present study with those of the research conducted in other fields.

Similar to the results of the present study, the findings of Rajabi ( 20 ), Abbasi ( 21 ), Rezaei ( 26 ) and Rahimniya et al. ( 30 ) show that the number of students in each field is a factor affecting the cost of students and increasing the number of students reduces the cost of education of each student. Also, similar to the results of the present study, Rajabi ( 20 ), Haghdoost and et al. ( 23 ), Ghasempour et al. ( 25 ) revealed that in higher educational levels, the costs of students increase.

In addition, Table 5 compares the budget paid by the government per student to study at each level with the cost of student education. As you see, the cost in all fields is more than the per capita cost of student education paid to the university. This result is consistent with those of Rezaei ( 26 ), Rahimniya et al. ( 30 ), and Kojuri et al.’s ( 31 ) studies. It is necessary to allocate the funds according to the type of university in the country, be more careful in allocating the funds, and determine the share based on per capita student costs; all universities and educational institutions operating in the education system should not be considered the same ( 31 ).

One of the limitations of the present study is the lack of access to information regarding the expenses incurred in the Ministry of Health and Medical Education. In addition, accounting information was recorded in the school by traditional cost systems and lacked a complete information about the ABC activities. This deficiency made it difficult to distinguish between information on expenses, except for salary information that was obtained directly from the payroll system. Nevertheless, every effort was made to ensure that the validity and reliability of this research are maintained as much as possible.

## Conclusion

The most important achievement and importance of the present study is to provide a model for calculating the cost of student education in the health sector using the ABC method. The significance of this study is that we can provide important and empirical evidence regarding the cost of medical school courses at different degrees in the largest medical universities in the country and compare them. In addition, it illustrated the steps on how to implement ABC in the health sector in order to demonstrate the usefulness of the ABC system and reduce the gap between theory and practice. This information can be used by health managers and decision makers to make the most of the budget, save money, and improve productivity.

As the results in Table 5 show, for non-clinical disciplines, on the average, the costs of training and the costs of overhead are equal, and each makes up half of the total costs. It seems that the reason is that the duration of these courses is long; as a result, the overhead costs in these courses have increased. On the other hand, these disciplines are less specialized and the salaries and benefits of its professors are less than clinical disciplines; therefore, the cost of training in these disciplines is lower than clinical disciplines. These two cases led to equalization of the costs of training and the costs of overhead in these disciplines.

For clinical fields, the costs of training is 77% and those of overhead is 23% of the total costs. In these fields, due to the fact that the length of the fields is shorter than non-clinical fields, the overhead costs is the average less than non-clinical fields. On the other hand, because the amount of tuition fees for professors in these fields is higher, the cost of training is higher than non-clinical fields. Therefore, in order to reduce the cost of students’ education, it is suggested that clinical officials should pay more attention to the efficiency of curriculum planning.

In general, 70% of the total costs of the education is the costs of student training and 30% of it belongs to the overhead costs. Based on the findings of the present study, it seems that the authorities should focus on controlling and reducing the costs of training, which is the main component of the costs. The results show that the more specialized the field, the higher the costs of education because it needs to be taught by professors with higher expertise and consequently higher salaries. Therefore, in these fields, the need for special attention of officials to the educational planning of students becomes more and more clear. In addition, given that non-faculty professors are paid less than faculty members, it is recommended that non-faculty professors should be used to reduce the costs of education for less sensitive and less specialized courses, provided that the quality of education is maintained.

It is also suggested that officials should pay attention to the costs of overhead. Because the main purpose of the students’ education is training, and the overhead costs provide less added value than the costs of training. On the other hand, reducing the costs of training, given the factors such as salaries and benefits of faculty members, the number of students in each field, etc. is hardly possible; hence, the importance of attempts to reduce the overhead costs becomes more apparent. In this regard, it is suggested that the officials of the headquarters and the medical school should determine the optimal number of manpower required for each department and review the arrangement of the staff by employing human resource management methods such as job measurement and timing.
